# Anesthetic management for upper arm amputation in a patient with acute rapid atrial fibrillation and a large thyroid goiter: A case report

**DOI:** 10.1097/MD.0000000000041254

**Published:** 2025-01-10

**Authors:** Yue Teng, He Wei, Siqi Hao, Yongshan Nan

**Affiliations:** a Department of Anesthesiology, Yanbian University, Yanji, Jilin, P.R. China; b Department of Anesthesiology, Beijing Friendship Hospital, Capital Medical University, Beijing, P.R. China; c Department of Anesthesiology, Yanbian University Hospital, Yanji, Jilin, P.R. China.

**Keywords:** a large thyroid goiter, a multi-regional block technique, atrial fibrillation, managing anesthesia

## Abstract

**Rationale:**

Patients with atrial fibrillation and a large goiter have high perioperative risks and often cannot tolerate general anesthesia, making it necessary for us to explore new safe and effective anesthesia methods.

**Patient concerns:**

The patient presented with atrial fibrillation accompanied by rapid ventricular rate, a thrombus attached to the left atrial appendage, and a massive thyroid goiter compressing the airway.

**Diagnosis:**

After the left humerus fracture surgery, the patient’s internal fixation loosened and fractured, accompanied by infection, formation of sinus tracts, and suppuration. Consequently, an emergency left upper arm amputation was performed.

**Interventions:**

An ultrasound-guided subclavian brachial plexus block combined with intercostobrachial nerve block via the axillary region was performed on a high-risk elderly patient.

**Outcomes:**

We opted for a multi-regional block technique, which allowed us to avoid the numerous risks associated with general anesthesia. The surgery proceeded smoothly, the patient reported no significant discomfort, and was discharged 1 week postoperatively.

**Lessons:**

This case demonstrates that a well-executed multi-regional block can provide satisfactory anesthesia, offering a viable alternative for managing anesthesia in high-risk patients.

## 1. Introduction

Atrial fibrillation (AF), the most common complex arrhythmia, has an increasing incidence with age. The primary type is paroxysmal atrial fibrillation, characterized by acute episodes lasting ≤ 7 days, which can often be resolved with medical intervention or spontaneously. AF is closely associated with various postoperative complications during the peri-anesthetic period, negatively impacting long-term prognosis. In severe cases, intra-atrial thrombus formation can occur, with emboli potentially causing systemic arterial embolism, notably cerebral embolism, which can be fatal.^[1]^ This report discusses a patient with AF, a large thyroid goiter, and the risk of intra-atrial thrombus embolization. The patient was admitted urgently for an amputation surgery due to an infection. The numerous risk factors present posed significant challenges for anesthesia and perioperative management.^[2,3]^

## 2. Ethics

This report has been approved by the Ethics Committee of the Yanbian University Hospital in China. The patient and the patient’s family have provided written informed consent for this report, and their information has been anonymized.

## 3. Case report

### 3.1. Patient characteristics

**Figure 1.**
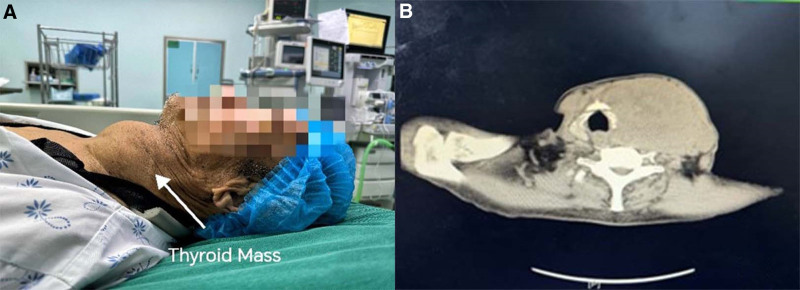
Patient imaging data. (A) Schematic diagram of the patient’s thyroid mass. (B) Chest computed tomography scan showing significant enlargement of the left lobe of the thyroid gland, measuring 110 × 70 mm in size.

The patient, a 79-year-old male, weighing 60 kg, with a height of 165 cm, and a BMI of 22.04 kg/m², was classified as ASA III. He was admitted urgently to Yanbian University Hospital on March 28, 2024, with a chief complaint of redness, swelling, heat, and pain with limited mobility in the distal left upper arm for 10 days, accompanied by exudation for 4 days. Thirty years ago, he underwent open reduction and internal fixation for a left humeral fracture at a local hospital. He has a history of arrhythmia, and an ECG showed ectopic rhythm, atrial fibrillation with rapid ventricular response, and left ventricular hypertrophy. Echocardiography revealed left atrial enlargement, mild pulmonary hypertension, reduced left ventricular systolic function, mild mitral regurgitation, mild tricuspid regurgitation, and a possible left atrial thrombus. Transesophageal echocardiography showed spontaneous echo contrast in the left atrial appendage, suggesting a thrombus. He also has a 10-year history of thyroid nodules, with significant enlargement of the left thyroid lobe, a large solid mass measuring 110 × 70 mm, none of which have received systematic treatment. Upon admission, the patient’s physical examination revealed: a temperature of 36.5 °C, a heart rate of 130 beats/min, a respiratory rate of 18 breaths/min, and blood pressure of 168/95 mm Hg. A specialized examination detected wheezing in the supraclavicular fossa, caused by the tracheal compression due to the thyroid mass, leading to the patient’s wheezing. Chest CT showed: symmetrical thorax, centered mediastinum, tracheal compression, and narrowing with rightward deviation, scattered patchy, and strip-like dense shadows in both lungs, and a significantly enlarged left thyroid lobe measuring 110 × 70 mm with multiple patchy low-density areas. Preoperative diagnoses included: (1) loosening and fracture of internal fixation device post left humeral fracture surgery; (2) nonunion of the left humeral fracture postsurgery; (3) radial nerve injury in the left upper arm; (4) left upper arm skin ulceration with infection (Fig. 1).

Due to the patient’s advanced age and the presence of a large thyroid goiter compressing the trachea, the trachea is narrowed and displaced to the right. If general anesthesia with muscle relaxants were used, there would be a risk of tracheal collapse leading to asphyxiation. Additionally, the patient has a mouth opening of <3 cm and is classified as Mallampati grade III, indicating a difficult airway and potential challenges with tracheal intubation. Upon admission, the initial diagnosis included atrial fibrillation with concurrent pulmonary infection. The patient’s overall condition was poor, making them unable to tolerate a second surgery. The internal fixation device had broken, accompanied by infection, and the development of a sinus tract with suppuration. It was crucial to control the ventricular rate promptly and proceed with the left upper arm amputation as soon as possible. The emergency surgery was scheduled for the first day after admission.

The patient did not experience significant symptoms of chest tightness or shortness of breath when lying supine or changing positions. Based on our years of clinical experience, we proposed a more suitable plan for the patient: using a combination of infraclavicular brachial plexus block and intercostobrachial nerve block via the axillary region, while preserving spontaneous breathing, to perform the left forearm amputation in the elderly patient. This approach completely avoids the challenges and perioperative airway management risks associated with tracheal intubation and general anesthesia. However, in practice, it is extremely challenging to meet the requirements of the anesthesia block range while maintaining stable intraoperative respiratory and circulatory conditions. This necessitates enhanced perioperative respiratory management and monitoring.

### 3.2. Anesthetic technique

After the patient entered the operating room, routine monitoring was initiated. The patient’s SpO_2_ was 92%, heart rate was 98 beats/min, respiratory rate was 20 breaths/min, and blood pressure was 159/90 mm Hg. Continuous arterial blood pressure monitoring was performed via right radial artery catheterization. The patient received oxygen via nasal cannula at a flow rate of 3 L/min. Right leg femoral vein catheterization was also performed. Under ultrasound guidance, a combined infraclavicular brachial plexus block and intercostal brachial nerve block through the axillary area were administered. Procedure details: the patient was positioned supine with arms naturally by the sides and head turned to the right. Intravenous sedation was provided with 1 mg of midazolam injection (Manufacturer: Jiangsu Enhua Pharmaceutical Group Co., Ltd., Approval No.: National Medicine Standard H19990027, Specification: 2 mL: 10 mg). The injection site was located at the intersection of the point 2 cm below the clavicle and the deltopectoral groove. After skin disinfection and sterile draping, local anesthesia was administered at the injection site. Ultrasound-guided nerve block localization was performed using a Wisonic portable ultrasound device (Manufacturer: China Huasheng Medical Technology Co., Ltd., China) equipped with a 5 to 10 MHz linear probe. The high-frequency linear probe was placed at the injection site to visualize the subclavian artery. The medial, posterior, and lateral cords of the brachial plexus were identified around the artery at the 3, 7, and 9 o’clock positions, with the pleura below. The puncture point was selected 2 cm proximal to the lower edge of the probe. Using the “in-plane” puncture technique, a disposable intravenous cannula was advanced under real-time ultrasound guidance until the brachial plexus was reached. After confirming no blood return on aspiration, 20 mL of 0.35% ropivacaine (Manufacturer: Qilu Pharmaceutical Co., Ltd., Approval No.: National Medicine Standard H20052690, Specification: 10 mL: 100 mg) was slowly injected.

**Figure 2.**
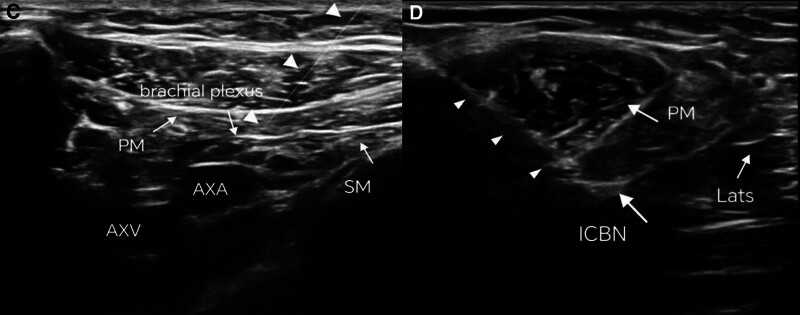
Schematic diagram of ultrasound-guided needle placement for nerve blocks. (C) Final ultrasound transducer position for ultrasound-guided subclavian perivascular brachial plexus block at the interscalene space, identifying the subclavian artery and visualizing the medial, posterior, and lateral cords of the brachial plexus, as well as the pleura beneath them, at 3, 7, and 9 o’clock positions relative to the artery. The “in-plane” approach is utilized for needle insertion. (D) Final ultrasound transducer position for ultrasound-guided intercostobrachial nerve block via the axillary region, with the head placed horizontally at the top of the axilla. The intercostobrachial nerve (ICBN) is visualized within the fatty layer posterior and superior to the axillary vein, between the lines connecting the edges of the teres major and latissimus dorsi muscles. AXA = axillary artery; AXV = axillary vein; ICBN = intercostobrachial nerve; Lats = latissimus dorsi muscle; PM = pectoralis major muscle; subclavius = subclavius muscle; white triangle indicates the trajectory of the needle insertion.

During the injection process, the needle tip direction and position were continuously adjusted until the gap was satisfactorily filled and enveloped by the fluid dark zone around the 3 bundles of the brachial plexus. After waiting for the anesthetic to take effect, the patient was able to abduct the upper limb, fully exposing the axillary area. The probe was placed transversely at the top of the axilla, adjusted to identify the axillary vein, and then moved medially towards the thoracic side of the axilla to visualize the teres major, latissimus dorsi, and axillary vein. The intercostobrachial nerve could be seen in the fat layer between the connecting line of the teres major and latissimus dorsi, superior–posterior to the axillary vein. Subsequently, 20 mL of 0.35% ropivacaine (manufacturer: Qilu Pharmaceutical Co., Ltd., approval number: H20052690, specification: 10 mL∶100 mg) was injected. Once the anesthetic took effect, the left forearm amputation surgery commenced. During the operation, the patient’s blood pressure and heart rate remained stable, and he reported no significant discomfort in the left limb. The surgery lasted 2 hours, with an intraoperative blood loss of 400 mL. The patient received 800 mL of crystalloid fluid and 400 mL of colloid fluid (Fig. 2).

The patient’s vital signs were stable, and he was alert and responsive. After 30 minutes of observation in the recovery room, he was comfortably transferred back to his ward. Postoperatively, patient-controlled analgesia was administered using sufentanil 100 µg and nalbuphine 40 mg. The patient experienced no phantom limb pain or limb ischemia–reperfusion injury postsurgery and was discharged 1 week later.

## 4. Discussion

In the above case, due to the presence of intracardiac thrombi caused by atrial fibrillation, it is necessary to minimize intraoperative hemodynamic fluctuations. Additionally, the presence of a large thyroid goiter poses significant challenges to the implementation of general anesthesia.^[3]^ In recent years, regional anesthesia techniques have become increasingly mature, and correct execution and appropriate use of regional anesthesia techniques can effectively provide postoperative analgesia and avoid hemodynamic fluctuations associated with general anesthesia.^[4]^ Therefore, for this patient, we chose the safer option of combining infraclavicular brachial plexus block with intercostobrachial nerve block via the axillary approach.

Under sedation, peripheral nerve blockade not only avoids the induction of general anesthesia and the hemodynamic changes commonly associated with it but also ensures adequate analgesia and reduces sympathetic nervous system tension in high-risk cardiac patients. With the widespread application of visualization techniques, ultrasound-guided nerve blockade has become a common anesthesia method in clinical practice, providing more precise and safer anesthesia effects for surgical procedures.^[5]^ Applying nerve blockade in surgery significantly reduces hemodynamic disturbances in patients, particularly beneficial for elderly and those with cardiopulmonary conditions. This technique minimizes potential organ damage, thus effectively mitigating anesthesia-related risks. In this case, due to obscured visualization of nerve and surrounding vascular tissues during brachial plexus block caused by a massive thyroid goiter obstructing the neck, and severe pain hindering adequate exposure of the axilla, ultrasound-guided supraclavicular brachial plexus block was chosen. Based on anatomical and innervation patterns, the medial cutaneous nerve of the arm is innervated by branches of the brachial plexus in addition to the intercostobrachial nerve.^[6]^ Currently, there is significant controversy regarding the origin of the intercostobrachial nerve. Recent studies suggest that the intercostobrachial nerve originates from the second intercostal nerve in 65.4% of cases, followed by a mix from the first intercostal nerve and the second intercostal nerve.^[7]^ The intercostobrachial nerve is purely a sensory nerve, primarily innervating the skin of the posterior upper arm, axilla, and lateral chest wall. Common methods for intercostobrachial nerve block include parasternal intercostobrachial nerve block and axillary intercostobrachial nerve block. The latter has been shown to provide more pronounced analgesic effects for upper arm surgeries.^[8]^

Therefore, during anesthesia solely with brachial plexus nerve block, incomplete coverage may lead to intraoperative patient discomfort, often necessitating additional analgesics to alleviate pain or even requiring a change in anesthesia method due to patient intolerance. Combining intercostobrachial nerve block via the axillary approach with brachial plexus nerve block can compensate for the inadequacy of isolated brachial plexus nerve block, providing patients with a more comfortable and safer anesthesia experience. However, there are few reports currently on the combined anesthesia of intercostobrachial nerve block with brachial plexus nerve block. Future studies may explore altering the block scope and expanding treatment windows to advance clinical research further.

Although this anesthesia method was successful in the perioperative management of a patient with acute rapid atrial fibrillation and a massive thyroid goiter requiring upper limb amputation, there are still some limitations. Firstly, no prospective randomized controlled study was conducted in this case, so sufficient evidence cannot be provided. Secondly, our current ultrasound equipment is unable to provide clearer images of anatomical structures, especially in patients with significant anatomical variations. Additionally, accurate localization of the needle tip during the puncture process is crucial. We are currently collecting more samples and hope to provide sufficient evidence through more prospective clinical trials in the future.

## 5. Conclusions

In summary, for patients encountered in clinical practice with acute rapid atrial fibrillation combined with a massive thyroid goiter requiring upper limb amputation, a combination of supraclavicular brachial plexus block and intercostobrachial nerve block anesthesia can be employed. During the procedure, close monitoring of hemodynamic changes in the patient is essential to ensure stability. Enhanced management and monitoring of the patient’s respiratory function during the perioperative period are also crucial to maintain airway patency. Additionally, vigilance is required to address intraoperative pain resulting from incomplete blockade due to anatomical variations. Furthermore, for elderly patients undergoing such procedures, adequate intraoperative warming measures should be implemented to prevent hypothermia. In conclusion, ultrasound-guided supraclavicular brachial plexus block combined with intercostobrachial nerve block provides excellent anesthesia efficacy in upper limb fracture surgery, resulting in high patient satisfaction and ensuring smooth surgical outcomes, thereby holding significant clinical application value.

## Acknowledgments

The authors would like to extend their heartfelt gratitude to Xianglan Jin and Huazhen Wang for their invaluable assistance in conducting and completing this research. Additionally, we are deeply appreciative of the cooperation from the Yanbian University Hospital.

## Author contributions

**Conceptualization:** Yue Teng.

**Data curation:** He Wei.

**Funding acquisition:** Yongshan Nan.

**Investigation:** Yue Teng.

**Methodology:** Siqi Hao.

**Writing – original draft:** Yue Teng.

**Writing – review & editing:** Yongshan Nan.
